# Assessment of the microvascular perfusion using sidestream dark‐field imaging in healthy newborn foals

**DOI:** 10.1002/vms3.1051

**Published:** 2022-12-16

**Authors:** Francesca Freccero, Chiara Di Maio, Jole Mariella, Aliai Lanci, Carolina Castagnetti, Gayle Hallowell

**Affiliations:** ^1^ Department of Veterinary Medical Sciences University of Bologna Ozzano dell'Emilia (BO) Italy; ^2^ Private Practitioner Italy; ^3^ Health Science and Technologies Interdepartmental Center for Industrial Research (HST‐ICIR) University of Bologna Bologna Italy; ^4^ IVC Evidensia, Pool House Equine Clinic Lichfield UK

**Keywords:** foals, microcirculation, oral mucosa, perfusion, SDF (sidestream dark‐field)

## Abstract

**Background:**

Different methods to measure tissue perfusion are available in equine neonatology, but they are not representative of microvascular derangements.

**Objective:**

To evaluate the feasibility of the sidestream dark‐field (SDF) capillaroscopy to visualize the capillary microvasculature in conscious newborn foals, the differences between two imaging sites and times of measurements, and the measurements' reproducibility.

**Methods:**

Seventeen healthy newborn foals were enrolled. Three sites at the upper and lower lip mucosa were assessed by SDF, using a hand‐held capiscope, at 24 h and at 4–5 days after birth. Videos were assessed independently by two observers for quality and for semiquantitative calculation of microvascular parameters, including vascular density (VD), microvascular flow index (MFI), proportion of perfused vessels (PPV), and functional capillary density (FCD). Data were analyzed using unpaired and paired Student's T‐tests to assess differences between sites and time‐points. Bland–Altman plots and intraclass correlation coefficient (ICC) were used to assess measurement reproducibility.

**Results:**

Differences were found between the upper and the lower lip for VD at both 24 h and 4–5 days, and for FCD at 24 h, and between the two time‐points for PPV at the lower lip. ICC for measurement reproducibility was good for all parameters (0.64–0.79) for the lower lip, and was good for VD and FCD (0,76–0,79) and fair to moderate for MFI and PPV (0.31–0.41) for the upper lip.

**Conclusions:**

Measurement of the capillary microvasculature is feasible in the conscious newborn foal. The lower lip has the best measurement reproducibility. Further investigations are warranted in cardiovascularly compromised cases, particularly in septic foals.

## Graphical abstract

This study evaluated the feasibility and reproducibility of sidestream dark‐field (SDF) imaging to visualize the capillary microvasculature in conscious newborn foals.

Seventeen healthy newborn foals were assessed on the upper and lower lip mucosa at 24 h and at 4–5 days after birth, and semiquantitative microvascular parameters calculated.

The SDF is feasible in the conscious newborn foal. The lower lip performed best with reproducibility; there were differences in microvascularization between sites and time‐points. This technique could detect microvascular changes in normal and cardiovascularly compromised foals.

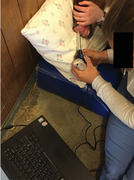

## 1 INTRODUCTION

The microcirculation is comprised of vessels with a diameter of less than 200 μm and is made up of arterioles (diameter 30 μm), capillaries (diameter 5–10 μm), and venules (50–100 μm). Microvessels less than 20 μm in diameter are essentially responsible for transporting oxygen, nutrients and play a key role in the clearance of waste products from tissues (Sjaastad et al., 2016).

The microcirculation is a main target of sepsis, which is one the most common presenting conditions in neonatal foals, along with perinatal asphyxia syndrome (Corley, 2002; Fielding and Magdesian, 2015). During sepsis, regulatory mechanisms of the cardiovascular system are impaired. Microcirculatory dysfunction is characterized by blood flow heterogeneity, with more perfused capillaries at the expense of less perfused ones (De Backer et al., 2010). Microcirculatory dysfunction may cause a reduction in tissue perfusion and if it progresses, microcirculatory pathology can result in organ failure in the face of adequate resuscitation maneuvers (Ince, 2005 ; Trzeciak & Rivers, 2005).

In equine neonatal intensive care, there are various indirect and non‐invasive methods used to monitor the global circulation and systemic hemodynamic status, including heart rate, capillary refill time, blood lactate concentration, indirect blood pressure, and urine output (Corley & Barr, 2018), some of which are extremely useful measures of end‐organ perfusion (Corley, 2003). However, all of these methods are poorly representative of microvascular function (Corley, 2002). Indeed, normalization of global hemodynamic parameters does not necessarily indicate that tissue perfusion and oxygenation are adequate (Palmer, 2014). Macrocirculation and microcirculation form a complex and dynamic physiological entity, and they are interdependent. Therefore, both components should be monitored and treated as needed (Schmid et al., 2013).

Sidestream dark‐field (SDF) imaging devices provide high contrast images of the microvasculature on a cutaneous or mucosal surface through a non‐invasive videomicroscope (Boerma et al., 2005; Massey & Shapiro, 2016). Microcirculatory structures can be observed in great detail: red blood cells are visualized as dark circulating bodies against a light background (Genzel‐Boroviczény et al., 2002). The SDF imaging device consists of a light guide surrounded by green light‐emitting diodes (LEDs; wavelength 530 nm) whose light penetrates the tissue and illuminates the microcirculation. The light is absorbed by hemoglobin of the red blood cells and scattered by leukocytes. A magnifying lens projects the image onto a video camera. Placed on organ (i.e., mucous membranes, skin, and serosae) surfaces, SDF imaging provides clear images of the red blood cells and leukocytes flowing through the microcirculation (Ince, 2005). Furthermore, by combining microscopic and photographic features, it allows for the generation of videoclips that show blood flow within the microvascular network and can be subsequently analyzed (Goedhart et al., 2007; Groner et al., 1999).

Various measurements used as markers of microvascular perfusion have been described by De Backer et al. (2007). These primarily include vascular density (VD), microcirculatory flow index (MFI), proportion of perfused vessels (PPV), and functional capillary density (FCD) or perfused vessel density (PVD).

Since the introduction of hand‐held SDF microscopy, microcirculatory abnormalities have been widely documented in sepsis and other diseases at the bedside of critically ill human patients and healthy and sick children and newborns, mostly using a sublingual site. (Trzeciak et al., 2007; Top et al., 2011; Alba‐Alejandre et al., 2013; González et al., 2017). The microcirculatory changes commonly identified in septic patients were considered as a prognostic index of mortality (De Backer et al., 2002). Additionally, these microcirculatory changes can be present in critically ill patients even when global perfusion is normal (Trzeciak & Rivers, 2005). A recent consensus has provided updated guidelines for use and interpretation of sublingual microcirculatory imaging (Ince et al., 2018).

In veterinary medicine, several studies have described the use of SDF and other similar technologies applied to oral mucosa in pigs (Erces et al., 2011; Guglielmi et al., 2004; Wester et al., 2011), dogs (Niemann et al., 2022; Silverstein et al., 2009; Silverstein et al., 2014), and cats (Goodnight et al., 2015; Yozova et al., 2022). In addition, microcirculatory imaging has been evaluated in conscious or anesthetized healthy and sick adult horses using the rectal, colonic, or oral mucosa, with some promising results for detecting microcirculatory alterations in different conditions (Croxford et al., 2013; Hallowell et al., 2013; Hurcombe et al., 2014; Kieffer et al., 2018; Mansour et al., 2021). However, the use of SDF imaging to evaluate the microcirculation has not been validated in conscious sick adult horses nor in foals.

The aims of this study are: to evaluate feasibility and measurement reliability of SDF imaging to assess the microvasculature in conscious healthy newborn foals, to investigate if measurements are affected by different sites and to investigate if there are detectable changes of the microvasculature in the early post‐natal life.

## 2 MATERIALS AND METHODS

### 2.1 Study population

This prospective observational study was performed on 17 healthy foals born to mares hospitalized for attended parturition at the Equine Perinatology Unit of the Department of Medical Veterinary Sciences, University of Bologna, during two foaling seasons (2017–2018).

Informed consent was given by the owners prior to enrollment. Inclusion criteria for foals were: normal parturition, APGAR score (Appearance, Pulse, Grimace, Attitude/Activity, Respiration) ≥ 8 at 5 min after birth (Vaala, 2006), normal complete blood count and serum biochemistry at birth, and IgG serum concentration >800 mg/dL at 18–24 h of life (Giguère and Polkes, 2005). At approximately 24 h, foals also needed to have a normal blood lactate concentrations around measured using a handheld lactate analyzer (Lactate Scout SensLab GmbH, Lepzig, Germany) and normal non‐invasive mean arterial blood pressure (MAP: 65–120 mmHg; Corley, 2004) using a cuff over the coccygeal artery (Dinamap Pro Series 300; Critikon Company L.L.C., Tampa, FL, USA) as described elsewhere (Giguère et al., 2005). Furthermore, they had a normal physical exam repeated at least twice daily during the observation period. Foals were free to nurse, housed in straw bedded boxes with their dams and turned out to pasture during the day.

### 2.2 Technique and procedure

Each foal underwent the microvascular imaging examination around 24 h (T0) and approximately 5 days (T5) after birth. A hand‐held SDF device (CapiScope® HVCS, KK Technology, Honiton, UK) composed of a probe and a digital video camera connected by a USB port to a laptop was used. One operator performed all the examinations. For each foal at each time‐point, SDF images were recorded at two sites on the oral mucosa (upper and lower lip). The foal was manually restrained in lateral recumbency on a soft mattress next to the mare. No sedation was used. Before acquisition, saliva was removed from the mucosa with a saline drenched gauze swab and the probe was covered with a disposable cap. The probe device was placed between the gum over the incisors and the lip mucosa (anterior vestibulum) avoiding excessive pressure, first on the upper and then on the lower lip (Supplementary Figure S1). For each foal, six cineloops were recorded at each time‐point, three at adjacent mucosal sites on the upper lip and three on the lower lip. Each cineloop was 5–10 s long (De Backer et al., 2007). The videos were converted into a .*avi* format and analyzed offline at the end of the collection period.

### 2.3 Video evaluation

To determine the inter‐observer measurement variability, videos from nine foals were analyzed independently by two observers blinded to foals. Initially, the two observers undertook a training phase which consisted of the analysis of ten randomly selected videos (not included in the analysis) followed by comparison and discussion of results. Thereafter, one of the observers (Obs.1) analyzed the video clips of the other eight foals (17 in total) applying the same criteria. Initially, each cineloop was evaluated for quality according to imaging quality criteria previously described in the literature (De Backer et al., 2007) (Table 1) assigned a score between 0 and 3. Video clips scored as “0” were discarded from further analysis.

**TABLE 1:** Prefixed score (0‐3) for qualitative assessment of quality of SDF videoclips, based on and modified from the criteria previously described by De Backer et al., 2007

3	No movements No pigmentation Absence of saliva and debris Very good focus Freeze frame more than 5 s Presence of visible capillaries in the same observational area
2	Little movement Little pigmentation Few areas with saliva and debris Good focus Freeze frame for 3–4 s Presence of visible capillaries in different observational areas
1	Some movement Some pigmentation Many areas with saliva and debris Adequate focus Freeze frame for 2–3 s Presence of visible capillaries in different observational areas
0	Significant movement A lot of pigmentation A lot of areas with saliva and debris Inadequate focus Freeze frame for 1 s or less Presence of pressure artifact

Semiquantitative evaluation and calculation of microvascular parameters included VD, MFI, PPV, and FCD, as described by De Backer et al. (2007). The VD was calculated as the number of <20 μm diameter vessels crossing three horizontal and three vertical lines divided by the total length of the lines. The MFI evaluated blood flow in small vessels (<20 μm) and was assessed qualitatively in four quadrants. A numeric classification was used to evaluate the flow in each quadrant: absent (0), intermittent (1), sluggish (2), or continuous (3). The mean of the four quadrants was then calculated to give MFI. The PPV measures the PPV compared with the total number of vessels in the field of observation (PPV (%) = (VD ‐ vessels with no/intermittent flow)/VD×100; De Backer et al., 2007). In this study, a simplified method to calculate PPV was used (PPV (%) = mean MFI/3×100; Hallowell, unpublished data). This is based on an indexed microvascular flow scoring used as an estimation for the proportion of microvessels with flow in the field. The FCD is an estimation of perfused capillary density compared with the total density of capillaries present in the field of observation and is calculated as a function of VD and PPV (FCD (1/mm) = VD × PPV) (De Backer et al., 2007).

### 2.4 Statistical analysis

Foals’ data were evaluated for normality using the Kolmogorov–Smirnov test. The mean of three measurements of each parameter (VD, MFI, PPV, and FCD) was calculated for the upper and lower lip. As all data were normally distributed, microvascular parameters were presented as mean ± standard deviation.

Bland–Altman plots and intraclass correlation coefficients (ICC) were used to evaluate measurement reproducibility between the two observers analyzing the images. Each observer undertook measurements in nine foals from both the upper and lower lip for all parameters. Measurements obtained at the two time‐points have been pooled together for each of the observers. ICC values below 0.50 were considered to show poor agreement, values between 0.50 and 0.75, 0.75 and 0.90 and above 0.90 were considered to reflect moderate, good, and excellent agreement, respectively (Koo and Li, 2016).

Unpaired and paired Student's T‐tests were used to identify differences in the microvascular parameters between sites and between time‐points measured by one observer (Obs.1) in the overall foals’ population (*n* = 17). Data were analyzed using an add‐in software package for Excel (Analyse‐it; version 2.03) and IBM SPSS Statistics 25 (IBM Corporation, Milan, Italy). The difference was considered significant when *p*<0.05.

## 3 RESULTS

### 3.1 Animals

Data from seventeen foals (11 females; 6 males) were included in this study. The mean weight was 48 ± 6 kg and APGAR score was 9 ± 1. The foals were aged 20 ± 6 h at initial evaluation (T0). At initial evaluation (T0) the heart rate was 98 ± 17 beats per minute, rectal temperature was 38 ± 0.4°C and indirect mean blood pressure was 78 ± 11 mmHg. Blood lactate concentrations were 2.5 ± 1.1 mmol/L, packed cell volume was 47 ± 4%, red blood cell count was 11.0 ± 0.87×10^12^/L and haemoglobin concentration was 15.6 ± 1.1 g/dl.

### 3.2 Technique feasibility and measurements reproducibility

In most foals, it was possible to achieve a complete examination in 15–30 min. Keeping the foal quiet and avoiding movements to acquire good quality cineloops was critical. For each foal, at least 12 cineloops were recorded, and a total of 204 cineloops were evaluated. Twenty‐four of the 204 videos (11.8%) were scored “0” and not analyzed. In total, 88 videos were analyzed at T0 (43 for the lower lip and 45 for the upper lip), and 92 videos at T5 (46 for the lower lip and 46 for the upper lip).

**FIGURE 1:**
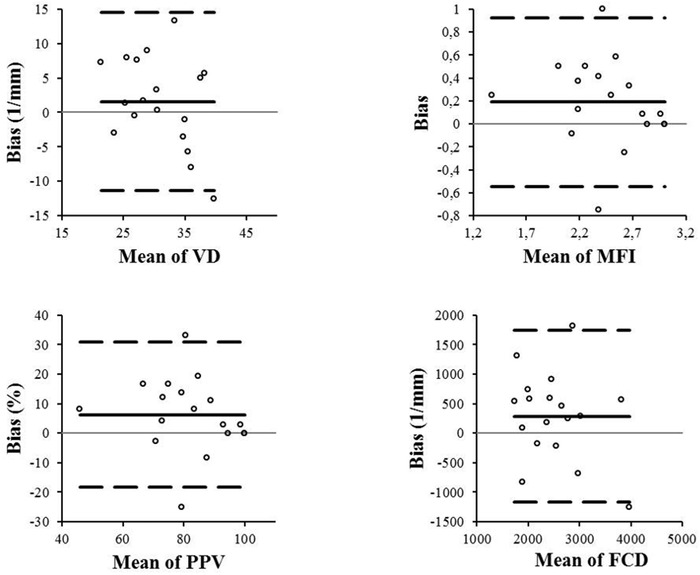
Bland–Altman plots for comparison between the two observers of all microvascular parameters in the lower lip. The solid line represents the bias (mean difference between two observers) and the two dotted lines the 95% limits of agreement (mean ± 1.96 SD) VD: Vascular Density; MFI: Microvascular Flow Index; PPV: Proportion of Perfused Vessels, FCD: Functional Capillary Density

**FIGURE 2:**
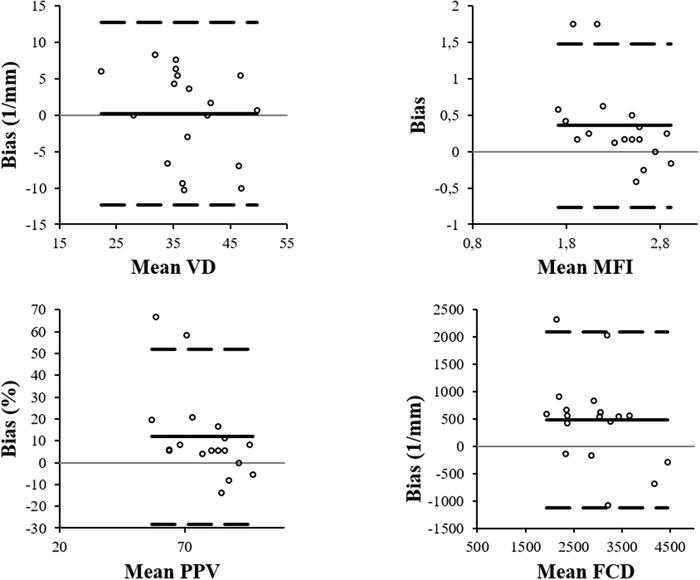
Bland–Altman plots for comparison between the two observers of all microvascular parameters in the upper lip. The solid line represents the bias (mean difference between two observers) and the two dotted lines the 95% limits of agreement (mean ± 1.96 SD). VD: Vascular Density; MFI: Microvascular Flow Index; PPV: Proportion of Perfused Vessels, FCD: Functional Capillary Density

Bland–Altman plots of interobserver measurement agreement for the upper lip are shown in Figure 1, for the lower lip in Figure 2. Their corresponding bias (95% limits of agreement) for VD, MFI, PPV, and FCD were 0.2 (0‐3.4), 0.4 (0‐0.6), 11.9 (11.7‐22.1), and 482 (74‐890), respectively, on the upper lip, and they were 1.6 (0–4.8), 0.2 (0–0.4), 6.3 (0.1–12.5), and 288.9 (0–659), respectively, on the lower lip.

For the upper lip, the ICC (95% confidence interval) for each microvascular parameter was: VD 0.79 (0.43–0.92), MFI 0.41 (0–0.78), PPV 0.31 (0–0.75), and FCD 0.66 (0.09–0.88). For the lower lip, the ICC was: VD 0.64 (0.04–0.87), MFI 0.78 (0.41–0.92), PPV 0.79 (0.44–0.92), and FCD 0.67 (0.12–0.88).

### 3.3 Comparison between upper and lower lip

Mean values obtained for the perfusion parameters at the two sites and time‐points in the overall population are reported in Table 2. The VD was higher on the upper lip respect to the lower lip both at T0 (*p* = 0.004) and at T5 (*p* = 0.04): VD values at the upper and lower lip were 40.2 ± 7.6 vessels/mm and 32.0 ± 7.6 vessels/mm at T0 and 40.2 ± 14.3 vessels/mm and 32.5 ± 11.1 vessels/mm at T5, respectively. There was a tendency for FCD to be slightly higher (*p* = 0.05) in the upper than in the lower lip at T0.

**TABLE 2:** Mean ± SD of the measurements of SDF microvascular perfusion parameters obtained by one observer at the upper and lower lip in 17 healthy foals within 24 h (T0) and at 5 days of life (T5)

	Upper lip		Lower lip	
	T0	T5	T0	T5
Vascular density (VD) (vessels/mm)	40.2 ± 7.6**	40.2 ± 14.3*	32.0 ± 7.6**	32.5 ± 11.1*
Microvascular flow index (MFI)	2.1 ± 0.6	2.2 ± 0.5	2.0 ± 0.5	2.3 ± 0.6
Proportion of perfused vessels	69.6 ± 19.0	72.2 ± 15.1	65.6 ± 17.5^§^	88.7 ± 29.8^§^
Functional capillary density (FCD) (vessels/mm)	2802 ± 1020	2621 ± 788	2062 ± 915	2479 ± 73

* The presence of a statistically significant difference between the upper and the lower lip (*p*<0.05)

** The presence of a statistically significant difference between the upper and the lower lip (*p*<0.01)

^§^ The presence of a statistically significant difference between the different time‐points (*p*<0.05)

### 3.4 Comparison between time‐points

The PPV measured on the lower lip was higher at T5 (88.7 ± 29.8%) than at T0 (65.6 ± 17.5%) (*p* = 0.03). No other significant time effect was found in any other perfusion parameter.

## 4 DISCUSSION

This study reports the successful use of SDF imaging to evaluate the microcirculation measured using the oral mucosa in healthy neonatal foals. Perfusion parameters showed overall satisfactory interobserver measurement reproducibility and allowed detection of some differences at different sites during the first few days of life.

Microvascular abnormalities can frequently be observed in critically ill patients and may play an important role in the pathogenesis of organ dysfunction (De Backer et al., 2010). In equine neonatal intensive care, methods to monitor the global circulation and systemic hemodynamic status are poorly representative of microvascular function (Corley, 2002), and microvascular alterations can persist despite correction of systemic hemodynamic variables (Palmer et al., 2014). Monitoring of the microcirculation over time may provide insights regarding the mechanisms and/or causes of shock (Elbers and Ince, 2006). Hand‐held vital microscopy (i.e., SDF) may help in certain circumstances such as in those foals that show persistent signs of hypoperfusion (e.g., lactate, urinary output) in the face of acceptable global hemodynamic parameters. During resuscitation or vasopressor therapy, it may help determining if tissue perfusion has improved (Ince, 2015), and be used to guide further treatment.

In this study, healthy subjects were utilized to first introduce and help establish a protocol for the use of SDF imaging in neonatal foals. The oral mucosa on the lips was chosen as the site for SDF assessment and proved to be easily accessible and well tolerated in this population of neonatal foals. While it was used in adult horses (Hallowell et al., 2013), the rectal mucosa was not considered a suitable site in foals, due to the size of the videomicroscope compared with the anal sphincter, foals’ movement, and peristaltic activity. Recently, the microcirculation has been assessed at the sublingual site in anesthetized horses during surgery (Mansour et al., 2021). However, the sublingual site was also not well tolerated in conscious foals. In human newborns, most of the studies used the sublingual and oral mucosa as sites of imaging (Boerma et al., 2005), since the same embryologic origin between oral and splanchnic mucosa has been demonstrated and thus may provide information about gastro‐intestinal perfusion (Creuteur et al., 2006). Notably, Hopster et al. (2018) reported that the oral mucosal blood flow assessed by laser Doppler flowmetry reflected changes of intestinal microcirculation in anesthetized horses. This may be relevant as a potential clinical application of SDF imaging in sick horses and foals. In addition, alterations in the renal microcirculation correlated with SDF measurements at the oral mucosa during sepsis and septic shock in a rat model (Hua et al., 2018).

The technique is simple and relatively fast to perform provided the foals remain still and enough people were available to assist with the procedure. In a previous study in healthy dogs, the videos were recorded at the oral mucosa under general anesthesia because it is acknowledged that patient movement interferes with image quality (Silverstein et al., 2009). Similarly, SDF imaging at the oral mucosa was described in anaesthetized horses undergoing surgery (Mansour et al., 2021). Indeed, the foal's movements represented a critical point to achieve good quality images in an acceptable recording time. It is worth noting that the SDF imaging could be of most interest in critically ill, septic foals. Most of these foals are usually less than one week‐old and often not very active and recumbent, due to their disease process. Such conditions may operatively facilitate the application of the technique.

It was essential to optimize the image obtained in real‐time for later microvascular flow analysis as image quality and ease of image analysis is influenced by several factors which include brightness, focus, image content, motion artifacts, and pressure (Massey & Shapiro, 2016, Ince et al., 2018). The use of the equipment required training and some practice, as excessive pressure occludes the circulation in the smaller vessels, making the images impossible to analyze, but could artificially reduce microvascular flow indices. The impact of pressure artifacts on microvascular flow assessed by SDF has been described in a pig model (Magnin et al., 2020). In this study, an inexperienced operator swiftly learned to acquire videos of adequate quality. The main quality requirement is that single red blood cells can be visualized in the capillaries, according to recent human guidelines (Ince et al., 2018). An objective scoring system to rate image quality is recommended as part of the analysis methods in the same guidelines (Ince et al., 2018), and the one described by De Backer et al. (2007) was used in this study.

In this study, the four parameters as described in the past consensus by De Backer et al. (2007) and an offline manual analysis (i.e., grid‐based) were employed to assess the microcirculatory pattern: VD, MFI, PPV, and FCD (Ince et al., 2018).

Measurement reproducibility of the perfusion parameters was evaluated between two observers. As previously reported in conscious sedated horses and dogs under general anesthesia (Silverstein et al., 2009; Hallowell et al., 2013), the measurement reproducibility from the oral mucosa resulted in overall satisfactory results. In foals, reproducibility was better for the lower (i.e., ICC moderate to good for the four parameters) than for the upper lip (i.e., poor to good for the four parameters). As previously noted, it is likely related to superior quality images obtained in this region (Hallowell et al., 2013). It is the authors’ impression that this might be due to a slightly more convenient position for the operator holding the probe at this level in the unsedated foal. For future studies and use in clinical cases, the lower lip should be selected for obtaining these images, based on findings in this study. Furthermore, the MFI and PPV performed best in terms of interrater reproducibility. In human studies, data on microcirculatory alterations are predominantly expressed in MFI and PPV. In particular, point‐of‐care analysis is mainly focused on MFI, as this parameter can be more easily assessed by visual inspection of the microvascular images (Ince et al., 2018). Moreover, in human critical care, a cut‐off for MFI has been suggested to discriminate clinically relevant microvascular alterations. (Ince et al., 2018). It may be advisable to focus on these parameters for future applications also in foals.

In terms of values, microvascular perfusion findings are difficult to compare across studies, due to differences in species, settings, and analytical methods. Vessel density at the oral mucosa was slightly greater in foals (regardless site and time‐point) when compared with values in healthy adult anaesthetized dogs (VD: median 24 vessels/mm), while FCD was similar (referred as to Perfused Vessel Density ‐ PVD: 2400 (1700‐3000) vessels/mm) (Silverstein et al., 2009). A slightly different measure of vessel density (referred as to Total Vessel Density ‐ TVD (mm/mm^2^) and derived parameters (Perfused Vessel Density ‐ PVD (mm/mm^2^) has been used in other equine studies (Kieffer et al., 2018; Mansour et al., 2021). The MFI and PPV are the perfusion parameters more consistently used across studies (Silverstein et al., 2009; Kieffer et al., 2018; Mansour et al., 2021). Mean MFI and PPV in foals were similar to adults anesthetized horses in one study (MFI median: 1.7 a.u.; PPV median: 70%; Mansour et al., 2021) while both MFI and PPV were lower than in anaesthetized dogs (MFI median: 3 a.u.; PPV median: 100%; Silverstein et al., 2009). The difference in VD may be a true anatomical difference, whereas the lower PPV, when compared with dogs may be a real difference in flow or possibly artefactual due to the additional challenges of obtaining diagnostic images in conscious healthy foals compared with anaesthetized animals. Furthermore, it should be mentioned that employing different methods to calculate PPV could have potentially contributed to the difference in PPV values compared with other studies.

When two sites of assessment were compared in this population of healthy foals, the upper lip yielded a greater VD than the lower lip. This could reflect an anatomical difference in the distribution of capillaries in the lips or could be artifactual, due to technical factors affecting imaging quality at different sites.

The only measure that differed over time was PPV measured at the lower lip which was higher at 5 days of life than at 24 h of age. PPV is an estimate of perfusion (flow) and not the number of capillaries in the microvasculature at the site of evaluation. The increase might be an effect of the cardiovascular adaptation taking place in the first days after birth in foals (Marr, 2015) or may relate to better quality images obtained where fewer vessels are compressed. Our results cannot be directly compared with other studies in horses or other species, as no similar conditions (i.e., species, age, timing, site, and protocol) have been investigated. In human medicine, it was demonstrated that the FCD measured in the buccal mucosa was higher in the first week of life than later in healthy neonates (Top et al., 2011), and that may be related to the notably higher cardiac output at this time (Stopfkuchen, 1987).

This study has several limitations. The videos were always recorded by one operator, thus the technique reproducibility could not be evaluated. Similar to other imaging modalities, the SDF imaging is substantially operator dependent, and the reliability of perfusion parameters when images are acquired by multiple operators should be tested, in view of a potential clinical application. Technique reproducibility was average to good for the same variables investigated at the oral mucosa in a previous study (Hallowell et al., 2013). This is in contrast to an experimental human study wherely, SDF microcirculatory parameters showed low intra‐rater and inter‐rater reproducibility (Valerio et al., 2019). In the latter study, the need of standardized conditions and a large sample size was remarked when attempting to detect clinically relevant intra‐individual differences (Valerio et al., 2019). This highlights that further work in more healthy and sick foals is warranted. In addition, as technique repeatability also was not evaluated, any differences between time‐points may be explained by technique variation rather than true differences.

The fact that the vessel density and flow (i.e., VD and MFI, and thus derived parameters) were manually (subjectively) assessed might also represent another limitation, although this was originally described by De Backer et al. (2007) and has been reported in other studies. Automatic vascular analysis software has been introduced also in equine medicine (Kieffer et al., 2018; Mansour et al., 2021) and eased the analysis respect to manually drawn grids used in this study. However, they both remain time consuming, and new technological developments will be directed to automatic real‐time assessment (Ince et al., 2018).

Rapid, bedside methods of microcirculation assessment, possibly automated by software, would be critical to introduce hand‐held vital microscopy also in equine clinical practice. Real‐time visual assessment by means of qualitative scoring system has proved good agreement with offline analysis of microcirculatory patterns in human patients (Ince et al., 2018), and has proven feasible in dogs (Gommeren et al., 2014). A prompt recognition and monitoring of microcirculatory alterations could potentially help with clinical decision making in neonatal critical care. Furthermore, as hemodynamic response to therapy needs ongoing re‐assessment, the possibility of single‐spot measurements for an extended time instead of multiple sites (De Backer et al., 2007) has been included in human guidelines (Ince et al., 2018), and should be pursued in future clinical studies in foals. Based on the present findings, measurement at the level of the lower lip would be suggested.

In conclusion, this study presents successful use of SDF imaging to estimate the oral microcirculation in healthy newborn foals. To our knowledge, this has not been previously described. The technique requires some practice, but proved to be feasible and well tolerated in this population of non‐sedated foals, and reproducible measurements of perfusion parameters could be obtained as reported in human patients and other species. Further studies are needed to determine age‐specific reference values in healthy foals, that could be used to define microcirculation‐guided therapy endpoints in critically ill foals. This technique could be a promising tool to assess the microcirculation alongside global circulation markers, and studies on its diagnostic and prognostic usefulness in critically ill and septic foals are justified. In view of a potential clinical application, rapid bedside microcirculation assessment methods, and single‐spot measurements should be pursued, and trials should focus on monitoring of microvascular parameters during specific therapeutical interventions.

## AUTHOR CONTRIBUTION

Francesca Freccero: Methodology; Resources; Writing – original draft; Writing – review & editing. Chiara Di Maio: Resources; Writing – original draft. Jole Mariella: Data curation; Writing – review & editing. Aliai Lanci: Methodology; Writing – review & editing. Carolina Castagnetti: Supervision; Writing – review & editing. Gayle Hallowell: Conceptualization; Methodology; Supervision; Writing – review & editing.

## ETHICS STATEMENT

The study has been approved by the ethics review committee at the University of Bologna. An informed client consent has been given for each animal.

## Supporting information


**Supplementary Figure 1**. Sidestream dark‐field imaging at the lip mucosa in a 24‐h‐old foal. The animal is positioned in lateral recumbency, the operator acquires the video clips by applying the probe perpendicularly to the mucosa of the inverted upper lip, exposed with the help of the staff.

## Data Availability

Full data are available on request.
