# Supplementary material for: Assessment of the microvascular perfusion using sidestream dark‐field imaging in healthy newborn foals
Source: Vet Med Sci. 2022 Dec 16;9(1):158–66. doi: 10.1002/vms3.1051 (PMC9857114; doi:10.1002/vms3.1051)
Supplement: Supplementary file 1 — Supplementary Figure 1. Sidestream dark‐field imaging at the lip mucosa in a 24‐h‐old foal. The animal is positioned in lateral recumbency, the operator acquires the video clips by applying the probe perpendicularly to the mucosa of the inverted upper lip, exposed with the help of the staff. [file VMS3-9-158-s001.docx]

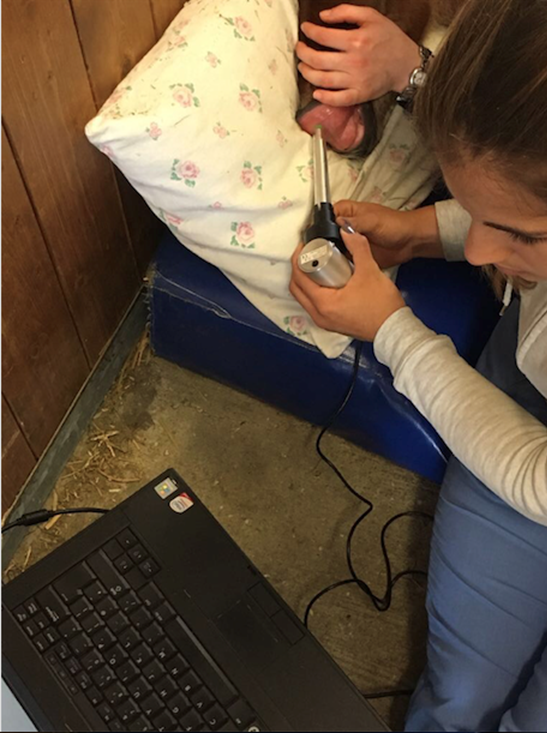


Supplementary Figure 1: Sidestream dark-field imaging at the lip mucosa in a 24-hour-old foal. The animal is positioned in lateral recumbency, the operator acquires the video clips by applying the probe perpendicularly to the mucosa of the inverted upper lip, exposed with the help of the staff.
